# Sarcoidosis With Pulmonary and Bone Marrow Involvement Following Chemotherapy in a Cancer Patient

**DOI:** 10.1002/rcr2.70353

**Published:** 2025-09-25

**Authors:** Ken Shirahase, Tadao Nagasaki, Masato Muraki, Tomoko Wakasa, Soichiro Hanada, Masamichi Iwai, Yuji Tohda, Hisako Matsumoto

**Affiliations:** ^1^ Department of Respiratory Medicine and Allergology Kindai University Nara Hospital Nara Japan; ^2^ Department of Respiratory Medicine and Allergology Kindai University Faculty of Medicine Osaka Japan; ^3^ Department of Pathology Kindai University Nara Hospital Nara Japan

**Keywords:** bone marrow, chemotherapy, pulmonary nodules, sarcoidosis

## Abstract

Sarcoidosis is a multisystem granulomatous disease that rarely involves the bone marrow and is particularly uncommon during chemotherapy. A 56‐year‐old Japanese woman with stage IIIc fallopian tube carcinoma developed persistent high‐grade fever and new bilateral pulmonary nodules two weeks after paclitaxel–carboplatin and bevacizumab therapy, which was complicated by neutropenia. Initial evaluations, including imaging, microbiological studies and bronchoscopy, excluded infection and malignancy recurrence. Histological analysis of bone marrow and transbronchial lung biopsy specimens demonstrated non‐caseating granulomas, confirming sarcoidosis despite normal serum angiotensin‐converting enzyme levels and absence of lymphadenopathy. Her fever and pulmonary nodules resolved spontaneously without corticosteroid therapy. This case underscores the importance of considering sarcoidosis in the differential diagnosis of fever and new pulmonary lesions in patients undergoing chemotherapy. Early histopathological confirmation is essential to avoid misdiagnosis and guide appropriate management.

## Graphical abstract

Patients undergoing chemotherapy who develop new pulmonary lesions accompanied by fever present a diagnostic challenge. Recurrence of malignancy, infection and inflammatory conditions must all be considered. We present a unique case of multi‐organ sarcoidosis with bone marrow involvement in a patient with fallopian tube carcinoma undergoing chemotherapy.

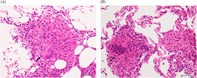

## 1 Introduction

Patients undergoing chemotherapy who develop new pulmonary lesions accompanied by fever present a diagnostic challenge. Recurrence of malignancy, infection and inflammatory conditions must all be considered. Excluding infection before resuming immunosuppressive therapy is vital, and definitive diagnosis typically requires tissue biopsy.

## 2 Case Report

**FIGURE 1:**
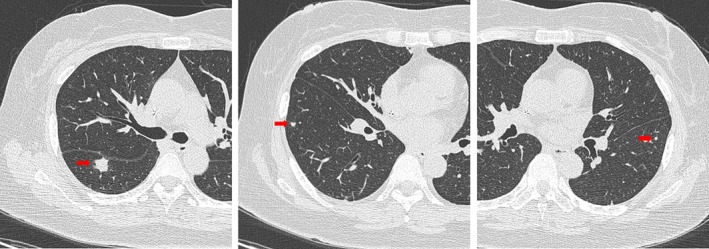
Chest computed tomography (CT) at admission showed numerous small bilateral pulmonary nodules up to 10 mm in diameter, without central necrosis, cavitation, or enlargement of mediastinal or hilar lymph nodes.

A 56‐year‐old Japanese woman with stage IIIc fallopian tube carcinoma was receiving adjuvant paclitaxel–carboplatin (and bevacizumab) chemotherapy via an indwelling central port. The patient had not received any immune checkpoint inhibitor therapy. About two weeks after her most recent chemotherapy, she developed a persistent fever without respiratory symptoms or history of travel or environmental exposures, including beryllium. On examination, she was febrile (40.2°C) and tachycardic (129/min) but remained normotensive with normal oxygen saturation on room air. Lung auscultation was clear. No lymphadenopathy or rash was noted. The chest port site had mild erythema and swelling. Laboratory tests showed elevated C‐reactive protein (12 mg/dL) with recovered neutrophils, and CA‐125 had risen to 77.4 U/mL from a normal baseline (Table 1). Chest CT revealed numerous bilateral pulmonary nodules up to 10 mm in diameter, without central necrosis or cavitation and no mediastinal or hilar lymphadenopathy (Figure 1). Abdominal CT did not reveal any significant abdominal lymphadenopathy. Transthoracic echocardiography revealed no valvular vegetations and no signs of basal interventricular septal thinning, wall motion abnormalities, or systolic dysfunction.

**TABLE 1:** Laboratory findings on admission.

WBC	3580/μL	Ca	8.1 mg/dL
Neut	61.7%	CRP	2.56 mg/dL
Eos	3.0%	Ferritin	555.5 ng/mL
Bas	0.7%	Procalcitonin	0.17 ng/mL
Lym	24.6%	sIL‐2R	3563 IU/mL
Mon	10.0%	CEA	2.3 ng/mL
RBC	3.41 × 10^6^/μL	CA19‐9	6.3 U/mL
Hb	9.9 g/dL	CA125	77.4 U/mL
Hct	31.0%	EBV‐VCA IgG	160 times
Plt	17.2 × 10^4^/μL	EBV‐VCA IgM	< 10
TP	5.6 g/dL	EBV‐EA IgG	< 10
Alb	3.2 g/dL	EBV‐EBNA IgG	80 times
T‐Bil	0.5 mg/dL	Anti‐MAC antibody	negative
AST	85 U/L	T‐SPOT.TB	negative
ALT	106 U/L	β‐D‐glucan	10.5 pg/mL
ALP‐JSCC	325 U/L	Cryptococcus antigen	negative
γ‐GT	198 U/L	Aspergillus antigen	negative
UA	3.2 mg/dL	Candida mannans antigen	negative
CK	10 U/L		
BUN	15.2 mg/dL	Arterial blood gas (room air)	
Cre	0.8 mg/dL	pH	7.391
Glu	109 mg/dL	PaCO_2_	41.1 Torr
Na	139 mmol/L	PaO_2_	111.0 Torr
K	4.2 mmol/L	HCO_3_^−^	24.4 mEq/L
Cl	105 mmol/L	Base Excess	0.0 mEq/L

Abbreviations: γ‐GT, gamma‐glutamyl transferase; Alb, albumin; ALP, alkaline phosphatase; ALT, alanine aminotransferase; AST, aspartate aminotransferase; Bas, basophil; BUN, blood urea nitrogen; Ca, calcium; CA125, cancer antigen 125; CA19‐9, carbohydrate antigen 19‐9; CEA, carcinoembryonic antigen; CK, creatine kinase; Cl, chloride; Cre, creatinine; CRP, C‐reactive protein; EA, early antigen; EBNA, Epstein‐Barr virus nuclear antigen; EBV, Epstein‐Barr virus; Eos, eosinophil; Glu, glucose; Hb, haemoglobinhemoglobin; HCO_3_^−^, bicarbonate; Hct, haematocrithematocrit; K, potassium; Lym, lymphocyte; MAC, *Mycobacterium avium* complex; Mon, monocyte; Na, sodium; Neut, neutrophil; PaCO_2_, partial pressure of arterial carbon dioxide; PaO_2_, partial pressure of arterial oxygen; pH, potential of hydrogen; Plt, platelet; RBC, red blood cell; sIL‐2R, soluble interleukin‐2 receptor; T‐bil, total bilirubin; TP, total protein; T‐SPOT.TB, T‐cell SPOT test for tuberculosis; UA, uric acid; VCA, viral capsid antigen; WBC, white blood cell.

Although empiric broad‐spectrum antibiotics were started and the central line was removed, the fever persisted and all cultures remained sterile. An initial bronchoscopy was nondiagnostic. A comprehensive infectious workup—including multiple blood and sputum cultures, fungal antigens, HIV and interferon‐gamma release assays, anti‐Mycobacterium *avium* complex (MAC) antibody testing, and Epstein–Barr virus serology indicating past infection—was uniformly negative. Serum angiotensin‐converting enzyme (ACE) was normal, whereas soluble IL‐2 receptor was elevated (3563 U/mL). Pulmonary function tests were within normal limits. Diffusion‐weighted whole‐body MRI showed uptake confined to lung nodules.

**FIGURE 2:**
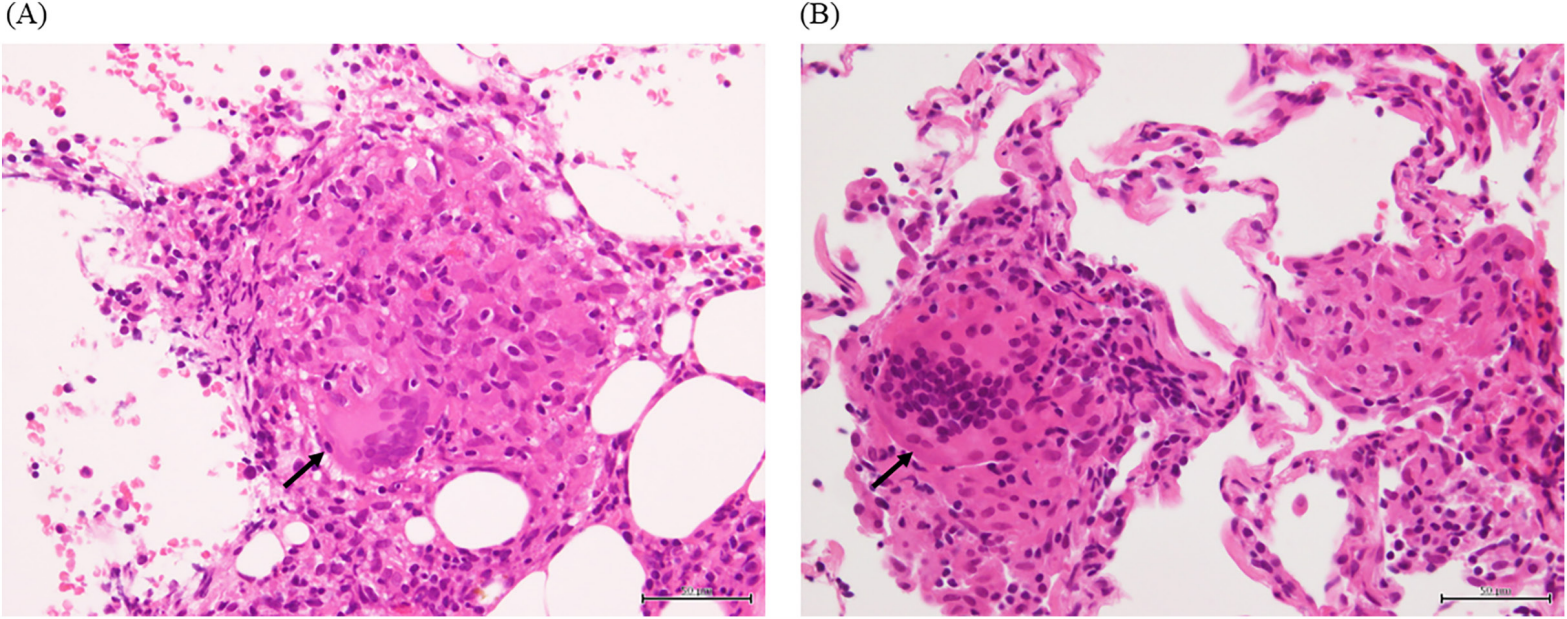
(A) Bone marrow biopsy demonstrated non‐caseating epithelioid granulomas in mildly hypocellular marrow with preserved trilineage haematopoiesis. No organisms were detected on Ziehl–Neelsen staining. Haematoxylin and eosin stain, original magnification ×200. (B) Histopathological examination of transbronchial lung biopsy specimens revealed non‐caseating epithelioid granulomas containing multinucleated giant cells. Haematoxylin and eosin stain, original magnification ×200.

On hospital day 10, the patient developed new pancytopenia; the complete blood count showed a white blood cell count of 2510/μL, haemoglobin 9.8 g/dL and platelet count 8.7 × 10^4^/μL. A bone marrow biopsy revealed multiple non‐caseating epithelioid granulomas and no malignant cells (Figure 2A). Special stains and cultures of the marrow for mycobacteria and fungi were negative. A second bronchoscopy showed lymphocyte‐predominant alveolitis (86%) with a CD4/CD8 ratio of 3.1 in bronchoalveolar lavage fluid, and random transbronchial biopsies demonstrated non‐caseating granulomas without organisms (Figure 2B).

A diagnosis of sarcoidosis involving both lung and bone marrow was made. We did not initiate systemic corticosteroid therapy since her symptoms were improving spontaneously and administering steroids would have increased infection risk. Chemotherapy had been paused during the diagnostic workup. Following discontinuation of chemotherapy, gradual haematologic recovery was observed without corticosteroid administration. On hospital day 26, the white blood cell count had increased to 4770/μL and the platelet count to 22.2 × 10^4^/μL, both of which remained stable without subsequent decline. Haemoglobin was 11.1 g/dL on day 26, which was low but had already been reduced since admission. Over subsequent months, her nodules regressed on follow‐up imaging and she remained asymptomatic without corticosteroid therapy. Subsequently, paclitaxel monotherapy was resumed. No recurrence of sarcoidosis was observed during follow‐up.

## 3 Discussion

Differentiating sarcoidosis from infection or metastatic disease in patients undergoing chemotherapy can be challenging. In our patient, the serum ACE level was within normal limits and imaging showed no hilar lymphadenopathy. A misdiagnosis could have resulted in inappropriate immunosuppressive therapy or unnecessary delays in chemotherapy.

Notably, sarcoidosis emerged during cytotoxic chemotherapy in this case. Given the immunosuppressive effects of chemotherapy, the emergence of an inflammatory granulomatous disease such as sarcoidosis is unexpected. Nevertheless, several reports describe sarcoidosis arising during or after chemotherapy. Paclitaxel–carboplatin therapy has been implicated in a few cases of sarcoidosis mimicking metastatic disease in ovarian and lung cancer patients, and granulomatous reactions have been observed after other regimens such as ABVD for Hodgkin lymphoma and FOLFOX for colorectal cancer in the literature [1, 2]. The exact pathophysiological mechanism linking chemotherapy to sarcoidosis remains unclear. Possible explanations include immune reconstitution phenomena following the chemotherapy‐induced nadir, unmasking of subclinical sarcoidosis due to reduced tumour‐related immunosuppression, or an exaggerated immune response to tumour antigens or cell debris released by chemotherapy‐induced cell death. Our case is unique in involving fallopian tube carcinoma and manifesting as multi‐organ sarcoidosis with bone marrow involvement, a rare presentation occurring in only 5%–10% of sarcoidosis cases [3].

Published cases of sarcoidosis arising during or shortly after cytotoxic chemotherapy have shown a heterogeneous course. Some patients improve with observation alone and do not require systemic therapy, whereas others are treated with corticosteroids. In contrast, reinitiation of the same cytotoxic regimen has rarely been documented. In one non‐Hodgkin lymphoma case, CHOP was continued and sarcoidosis resolved spontaneously [4]. In the present case, despite multi‐organ involvement, sarcoidosis resolved without the need for systemic corticosteroid therapy. Moreover, paclitaxel monotherapy was subsequently resumed without recurrence of sarcoidosis.

Although sarcoid‐like reactions associated with malignancy were considered, sarcoid‐like reactions typically localise to tumour‐draining lymph nodes and do not involve distant organs such as the bone marrow. Our patient's disseminated granulomas in the lung and marrow, along with her spontaneous improvement without cancer therapy, supported a diagnosis of true systemic sarcoidosis rather than a localised sarcoid reaction.

Additionally, miliary tuberculosis and disseminated MAC can cause high fevers with lung nodules and bone marrow granulomas. The granulomas were non‐caseating in this case; however, caseation can sometimes be minimal or absent in early disease [5]. Despite negative results on all tests for mycobacterial infection, including sputum cultures, histologic stains of biopsy specimens and the T‐SPOT.TB assay, definitively excluding tuberculosis was challenging because of Japan's relatively higher tuberculosis prevalence among developed countries. Ultimately, the absence of organisms on repeated cultures and stains, combined with atypical radiographic features, argued strongly against a mycobacterial infection.

In conclusion, fever and new pulmonary nodules in chemotherapy patients are not always attributable to infection or tumour relapse. Early histopathological confirmation is vital to establish the correct diagnosis and guide proper management, ensuring that both the malignancy and coexisting sarcoidosis are treated appropriately.

## Author Contributions

Tadao Nagasaki wrote the first draft. All the authors commented on earlier versions and approved the final manuscript.

## Consent

The authors declare that written informed consent was obtained for the publication of this manuscript and accompanying images using the form provided by the Journal.

## Conflicts of Interest

The authors declare no conflicts of interest.

## Data Availability

The data that support the findings of this study are available from the corresponding author upon reasonable request.
